# Human rhinovirus infection blocks SARS-CoV-2 replication within the respiratory epithelium: implications for COVID-19 epidemiology

**DOI:** 10.1093/infdis/jiab147

**Published:** 2021-03-23

**Authors:** Kieran Dee, Daniel M Goldfarb, Joanne Haney, Julien A R Amat, Vanessa Herder, Meredith Stewart, Agnieszka M Szemiel, Marc Baguelin, Pablo R Murcia

**Affiliations:** 1 MRC-University of Glasgow Centre for Virus Research, Glasgow, UK; 2 MRC-University of Glasgow Centre for Virus Research, Institute of Infection, Immunity and Inflammation, College of Medical, Veterinary and Life Sciences, University of Glasgow, Glasgow, UK; 3 Imperial College London, London, UK; 4 School of Veterinary Medicine, College of Medical, Veterinary and Life Sciences, University of Glasgow, Glasgo, UK

**Keywords:** SARS-CoV-2, Rhinovirus, Virus-virus interactions

## Abstract

Virus-virus interactions influence the epidemiology of respiratory infections. However, the impact of viruses causing upper respiratory infections on SARS-CoV-2 replication and transmission is currently unknown. Human rhinoviruses cause the common cold and are the most prevalent respiratory viruses of humans. Interactions between rhinoviruses and co-circulating respiratory viruses have been shown to shape virus epidemiology at the individual host and population level. Here, we examined the replication kinetics of SARS-CoV-2 in the human respiratory epithelium in the presence or absence of rhinovirus. We show that human rhinovirus triggers an interferon response that blocks SARS-CoV-2 replication. Mathematical simulations show that this virus-virus interaction is likely to have a population-wide effect as an increasing prevalence of rhinovirus will reduce the number of new COVID-19 cases.

**Footnote page**

PRM owns shares of Astra Zeneca. The rest of the authors declare no conflicts of interest.

This work was supported by grants from the Medical Research Council of the United Kingdom (MC_UU_12014/9 to PRM, MR/N013166/1 to JH and MR/R502327/1 to DMG). MB is supported by centre funding from MRC under a concordat with the UK Department for International Development and the National Institute for Health Research Health Protection Research Unit in Modelling Methodology. VH was funded by the German Research Foundation (Deutsche Forschungsgemeinschaft; project number 406109949) and the Federal Ministry of Food and Agriculture (BMEL; Förderkennzeichen: 01KI1723G). JARA is supported by the University of Glasgow School of Veterinary Medicine VetFund.

The information in this manuscript has not been presented at any conference or meeting.

## Background

The rapid spread of COVID-19 and its impact on global health highlights the importance of viral respiratory diseases. The human respiratory tract hosts a community of viruses that includes members of the *Orthomyxoviridae* (e.g., influenza virus A and B), *Pneumoviridae* (e.g., respiratory syncytial virus), *Picornaviridae* (e.g., rhinovirus), *Coronaviridae* (e.g., severe acute respiratory syndrome coronavirus 2) and others [1, 2]. We and others showed that interactions between co-circulating, taxonomically different respiratory viruses, can influence patterns of infection [3, 4]. We showed that human rhinoviruses (HRVs) and influenza A viruses (IAVs) interact negatively at the individual patient and population level. Additionally, it has been postulated that the circulation of HRV delayed the spread of pandemic H1N1 influenza virus in France in 2009 [5]. Viral interference interactions at the host level are considered important in influencing observed population dynamics. Wu et al. demonstrated that HRV induces an interferon (IFN) response that protects against subsequent IAV infection in differentiated airway cultures [4], whereas Gonzalez et al. showed that RV attenuates influenza severity in a mouse model [6].

Non-pharmacological interventions have hampered our ability to determine the impact of severe acute respiratory syndrome coronavirus 2 (SARS-CoV-2) on the epidemiology of respiratory viruses. However, it is possible that the emergence of SARS-CoV-2 will affect their ecology. Co-infection studies using air-liquid interface cultures of differentiated respiratory epithelial cells can shed light on the nature of SARS-CoV-2 interactions with other viruses and their effect on virus replication. Here, we examined the replication kinetics of SARS-CoV-2 in the presence of HRV in the human respiratory epithelium. HRV was selected due to i) its high prevalence in the human population [7]; ii) its negative interaction with IAV at the host and population level [3, 4]; iii) its ability to induce a strong IFN response [4]; and iv) the sensitivity of SARS-CoV-2 to IFN [8]. We used our experimental results as a proxy of within-host coinfection dynamics to simulate the impact of HRV circulation on the epidemiology of SARS-CoV-2 under different scenarios of HRV prevalence.

## Methods

### Cells

Primary human bronchial epithelial cells (HBEC) were sourced from Epithelix Sarl (Geneva, Switzerland). Cells were maintained and seeded on transwells (Cell culture inserts, Falcon® Cat. No.: 734-0036) using Epithelix hAEC media (Epithelix, EP09AM) and incubated at 37oC with 5% CO2. An air-liquid interface (ALI) was initiated once they reached confluency, when the maintenance media was switched to Pneumacult$^{TM}$-ALI media (Cat. No.: 05001, STEMCELL Technologies). Vero E6 F5 cells were subcloned from Vero E6 cells, which were a gift from Prof. Michele Bouloy. A bulk population of VeroE6 cells was diluted in DMEM supplemented with 10% (v/v) foetal calf serum to 1 cell per 100 ul and plated into a 96 well format and incubated at 37C in a 5% CO2, humidified incubator. Wells were assessed for cell number with 0 and 3 cells/well observed. Once the population had expanded, each clonal population was further seeded into a single well in a 96 well-plate. The next day, the plate was infected with 8400 pfu/well of SARS-CoV2 and left for 72 hours. The plates were fixed in 8% (w/v) formaldehyde in PBS and stained with Coomassie brilliant blue (0.1% [w/v] Coomassie Brilliant Blue R-250; 45% [v/v] methanol; 10% [v/v] glacial acetic acid) and assessed for cytopathic effect. Plates were scanned using a using the Celigo platform (Nexcelcom). Infection of 3 of 288 clones resulted in the clearance of the monolayer (2H6, 5F3 and 6F5). These clones were further assessed for changes in plaque morphology, and whether the well-clearance assay generated representative titers. They were further assessed for growth characteristics. Two of the three clones were discarded due and underestimate of virus titer (2H6) and longer mean generation time of the cells (5F2) in comparison the bulk population of VeroE6. HeLa Ohio cells were a gift from Dr. Toby Tuthill (The Pirbright Institute). Both cell lines were grown in Dulbecco's Minimum Essential Media (DMEM), high glucose, GlutaMAX supplemented with 10% fetal bovine serum (FBS) and 1% non-essential amino acids (NEAA).

Viruses

SARS-CoV-2 strain HCoV-19/England/02/2020 was sourced from Public Health England (GISAID accession: EPI_ISL_407073) originating from a clinical isolate and was passaged twice in VeroE6 cells. HRV-A16 was sourced from the American Type Culture Collection (ATCC) (ATCC VR-283).

Infection of HBEC cultures

Infection of HBECs. HBEC cultures were infected at ≥ 35 days post ALI initiation. The apical surface of the cultures was washed twice with serum free DMEM before infection (24 hours prior to infection and immediately before infection). Cells were inoculated with $10^4$ PFU of either SARS-CoV-2 or HRV-A16, or a mixture containing $10^4$ PFU of each virus and incubated at 37°C for 120 minutes. Previous experiments showed that inoculation of ALI cultures with 10,000 plaque forming units (PFU) resulted in consistent replication of HRV and SARS-CoV-2 [9]. The inoculum was removed, and cultures were washed once. This wash was titrated by 50% tissue culture infectious dose ($TCID_{50}$) assay and served as the 0-hour time point for growth curves. Cells were incubated at 37°C with 5% $CO_2$. At each time point, serum free DMEM was added apically to each culture and incubated for 30 minutes at 37°C. This was removed, aliquoted and stored at -80°C prior to subsequent titration. Each infection was carried out in two independent experiments and each experiment consisted of at least three technical replicates. Titrations of SARS-CoV-2 and HRV-A16 were performed on Vero E6 6F5 and HeLa OH cells, respectively. Virus samples were titrated in ten-fold serial dilutions in DMEM with 2% FBS and 1% NEAA on confluent monolayers of cells. Each sample was titrated in triplicate. SARS-CoV-2 $TCID_{50}$ plates were incubated at 37°C and HRV-A16 plates were incubated at 33°C. Plates were incubated for approximately 72 hours and fixed in 8% formaldehyde and stained with 0.1% Coomassie Brilliant Blue. Cytopathic effect was recorded, and a TCID50/ml titre determined as calculated by the Spearman and Kärber algorithm [10]. For BX795 experiments, ALI cultures were transferred to

Pneumacult$^{TM}$-ALI media containing 6 uM of BX795 (or DMSO) 18 hours prior to infection and media were changed daily. All experimental infections were carried out under Biosafety Level 3 conditions.

Tissue processing and immunostaining

After fixation in 8% formaldehyde for 16-24 hours, HBEC cultures were processed overnight for paraffin-embedding, sectioned to 2-3 µm-thick sections and mounted on glass slides. Two sections for each condition were sectioned and processed using pH 8 EDTA antigen retrieval and permeabilized with 1% triton. DAPI (Thermo Fisher Scientific, cat# P36392) was included in the mounting medium, and slides were stained with primary sheep anti-N (nucleocapsid) IgG antibody (DA114, mrcppu-covid.bio, 1: 1000 dilution), primary mouse anti-MxA antibody [11], primary mouse anti-VP2 antibody (QED Bioscience Ltd. – 18758), or a primary rabbit-anti-hACE2 (Cell Signalling Technology) antibody. For immunofluorescence, primary antibodies were detected using an AlexaFluor 555-conjugated donkey anti-sheep antibody (A11015, Thermo Fisher Scientific, 1:1000 dilution) and an AlexaFluor 488-conjugated goat anti-mouse (Sigma SAB4600056, 1:1000 dilution). For immunohistochemistry, anti-hACE2 was detected using EnVision+ anti-rabbit HRP (Agilent K4003). IF sections were imaged using a Zeiss LSM880 confocal microscope and IHC sections were imaged with an Olympus BX51 microscope.

Statistical analysis and data visualisation

Statistical analysis and data visualisation were carried out in R 3.5.1 [12]. Multivariable logistic regression models were used to investigate significance among the different conditions. Those models accounted for biological replicates as this parameter was uneven, as well as treatment, and time post-infection. When biological replicate was not a significant parameter, this latter was removed to simplify the model. Models were run using the lme4 package [13]. Data visualisation and figures were generated using ggplot2 package [14].

**Results**

To determine if SARS-CoV-2 and HRV interact within the human respiratory epithelium, we infected air-liquid interface (ALI)-cultures of human bronchial epithelial cells (HBECs) with either SARS-CoV-2, HRV or with both viruses simultaneously. To assess the impact of coinfections on the replication kinetics of each virus, HRV and SARS-CoV-2 titers were determined at different times post infection from apical washes of coinfected cells and compared to their respective titers from single virus infections. SARS-CoV-2 exhibited highly contrasting replication kinetics in single and coinfections (p= 0.03928, Figure 1A). SARS-CoV-2 titers increased slowly from 24 hours post-infection (hpi) onwards and up to 96 hpi in single infections, whereas in coinfections with HRV, SARS-CoV-2 titers decreased rapidly and were undetectable at 48 hpi (Figure 1A). In contrast, HRV titers displayed the same kinetics in single and coinfections: they increased rapidly during the first 24 hours, followed by a gradual and sustained decline (Figure 1B). As simultaneous coinfections might not occur frequently during natural infection, we performed staggered coinfections of ALI-cultures of HBECs as follows: cells were infected with HRV, and 24 hours later they were infected with SARS-CoV-2. This experiment was also repeated in the reverse order (i.e., SARS-CoV-2 first, followed by HRV). As observed in simultaneous coinfections, SARS-CoV-2 growth was severely impaired in both staggered coinfections: when SARS-CoV-2 inoculation was followed by HRV infection (p= 0.0260) SARS-CoV-2 replication increased between 24 and 48 hpi as seen in SARS-CoV-2 single infection, but a subsequent sharp decrease was observed between 48 and 96 hpi (Fig, 1C). When HRV inoculation was followed by SARS-CoV-2 infection, SARS-CoV-2 replication did not exceed the inoculum titer and viral titers quickly declined (p= 0.0063) (Figure 1D). In contrast, the growth of HRV was unaffected by SARS-CoV-2 (p= 0.2027) regardless of the sequence order of infections (Figure 1C and 1D). When SARS-CoV-2 was inoculated first, the growth curve of HRV shifted and peaked at 72 hpi (Figure 1C), reflecting the delay in HRV inoculation. We tested if the observed reduction of SARS-CoV-2 titers was due to a block in virus entry due to HRV-

induced downregulation of the SARS-CoV-2 receptor, ACE2 [15]. To this end, we used immunohistochemistry to detect ACE2 in HRV or SARS-CoV-2 single infected and coinfected epithelial cells. We observed high levels of ACE2 expression on the apical surface of the epithelium regardless of the infection status of the cells (Figure S1) suggesting that HRV blocks SARS-CoV-2 infection via mechanisms that are independent of virus entry.

SARS-CoV-2 is susceptible to IFN and encodes multiple genes that alter signaling pathways upstream and downstream of IFN production [8]. As HRV induces an interferon-mediated innate immune response that blocks IAV in ALI-cultures [4] we hypothesized that the observed block in SARS-CoV-2 replication was due to an HRV-triggered IFN response. To test this, we used fluorescence microscopy to examine the IFN-mediated innate immune activation induced by each virus. Specifically, we compared the *in situ* expression of MxA, a protein encoded by an IFN-stimulated gene that is highly upregulated upon IFN production [11]. Figure 2 shows that ALI-cultures of HBECs infected with HRV express high levels of MxA, contrasting with the low levels of MxA observed in SARS-CoV-2 infected cultures. Coinfected cultures exhibited high levels of MxA expression, similar to those exhibited in single infections with HRV (Figure 2). We further performed immunofluorescence using antibodies directed against the nucleocapsid (N) of SARS-CoV-2 and observed that N expression is clearly detected mainly on the apical area of epithelial cells subject to single SARS-CoV-2 infection, but undetectable in co-infected cells (Figure 3). Overall, our combined experiments confirmed i) that SARS-CoV-2 replication within the ALI-cultures of HBECs does not progress in the presence of HRV and ii) that HRV triggers a faster and likely stronger IFN response compared to SARS-CoV-2. We therefore hypothesized that the block observed in SARS-CoV-2 replication was due to an innate immune response triggered by HRV. To test this, we performed HRV/SARS-CoV-2 coinfections in the presence of BX795, an inhibitor of TANK-binding kinase 1 that has been shown to block the IFN-mediated innate immune response in differentiated cultures of respiratory epithelium [4]. In the presence of BX795, the ability of SARS-CoV-2 to replicate in the respiratory epithelium is restored to comparable levels to SARS-CoV-2 single infection, despite the presence of HRV (Figure 4A). This confirms that the observed block in SARS-CoV-2 replication in coinfections with HRV was the result of negative interactions driven by the innate immune response induced by HRV. Interestingly, HRV replication was also increased in the presence of BX795 and titers plateau between 48 and 96 hpi, rather than declining as observed in the DMSO control coinfection and HRV single infection (Fig 4B). This indicates that virus-induced innate immune signaling also hampers HRV replication in HBECs.

Given the high prevalence of HRV, we wanted to test if the observed within-host interference could have an impact on the number of new COVID-19 cases in the population. We performed mathematical simulations using the moment generating function equation [16] to derive the change in the growth rate of SARS-CoV-2 infections as a result from having a fraction of the population refractory to COVID-19 due to an episode of HRV infection (Data analysis S1 in Supplementary Material).  Our results show that the number of new SARS-CoV-2 infections decreases as the number of HRV infections increase, and this reduction increases with higher HRV prevalences and longer duration of the interference effect (Figure 5). When SARS-CoV-2 growth rates are low, HRV circulation can lead to SARS-CoV-2 infections not spreading, whereas exponential growth is expected in the absence of HRV.

## Discussion

Respiratory explants and ALI-cultures of human airway epithelium provide a highly controlled cellular environment that mimics to a considerable extent the natural site of infection and thus enables us to model the impact of virus tropism and innate immune responses on within-host infection dynamics [17]. Here we showed that HRV infection impairs SARS-CoV-2 replication and spread within the human respiratory epithelium. Our study shows that HRV exerts an indirect negative interaction, with a dominant inhibitory phenotype against SARS-CoV-2. Specifically, we showed that HRV triggers an IFN response that makes most cells nonpermissive to SARS-Cov-2 infection, while HRV is unaffected by the presence of SARS-

CoV-2. The susceptibility of SARS-CoV-2 to the IFN response is illustrated by the number of genes present in its genome that are devoted to overcome the innate immune response (reviewed in [18]). We also showed that HRV hampers SARS-CoV-2 replication even when the former was inoculated 24 hours after SARS-CoV-2. Overall, our results demonstrate that viral interference interactions induced by HRV infection can inhibit SARS-CoV-2 replication in the respiratory epithelium and builds on previous epidemiological, modelling, and experimental work on virus-virus interactions [3-5, 19]. Future studies to elucidate the molecular mechanisms of viral interference could enable us to wield virus-virus interactions to our advantage and use them as control strategies or therapeutic measures. For example, screening for HRV-induced genes with anti-SARS-CoV-2 activity might constitute a future research avenue to develop antiviral therapies against coronaviruses.

Recently, Wu et al. [4] showed that the IFN response triggered by HRV also interferes with IAV replication. Our combined studies suggest that viruses that stimulate an IFN response in the respiratory epithelium might interfere with SARS-CoV-2 and IAVs. These findings have important implications, as they suggest that immune-mediated effects induced by mild, common cold virus infections, including HRV, might confer some level of protection against SARS-CoV-2, potentially attenuating the severity of COVID-19. Given the high transmissibility and prevalence of HRV, this effect might have an impact on the disease burden caused by COVID-19 at the population scale, with expected heterogeneities depending on HRV prevalence among different age groups. For example, this interference effect can contribute to differences in SARS-CoV-2 transmission between school-aged children (with high prevalence of HRV) and adult populations (with comparatively lower HRV prevalence).

Viruses are obligate intracellular pathogens that can only infect a restricted number of cell types within the body (a property known as tropism). Virus-virus interactions are likely to occur not only in the respiratory tract but also in other tissues that support multi-virus environments, such as the gastrointestinal tract, where enteric infections are modulated by the gut virome [20] and also affect the immunogenicity of the live attenuated polio vaccine [21]. The nature of such interactions (i.e., positive, negative, or neutral) is largely unknown and likely to be influenced by the specific viruses involved, the timing of each infection and the interplay between the host's response to each virus.

There is a vast body of knowledge on the impact of evolution on virus-host interactions [22-25]. Many studies have focused on the evolutionary arms race between viruses and hosts, where the host's immune system evolves antiviral mechanisms to stop viral replication and viruses evolve to evade antiviral proteins. We propose that virus-virus interactions influence this arms race and contribute to shaping their molecular interplay. For example, it is feasible to think that HRV infections in humans might be mutually beneficial: from a HRV perspective, humans evolved a tightly regulated immune response that allows HRV to replicate and transmit while it blocks other potentially competing viruses. From a host's perspective, HRV infections, which are usually associated with mild disease, stimulate an antiviral response that prevents infections by more severe (and sometimes lethal) viruses such as SARS-CoV-2 and IAV. Future studies using co-infections are needed to shed light on the role of ecology and evolution on virus-virus interactions and their impact on virus host range, transmission and disease.

**Acknowledgments.** This work was supported by grants from the Medical Research Council of the United Kingdom (MC_UU_12014/9 to PRM, MR/N013166/1 to JH and MR/R502327/1 to DMG). MB is supported by centre funding from MRC under a concordat with the UK Department for International Development and the National Institute for Health Research Health Protection Research Unit in Modelling Methodology. VH was funded by the German Research Foundation (Deutsche Forschungsgemeinschaft; project number 406109949) and the Federal Ministry of Food and Agriculture (BMEL; Förderkennzeichen: 01KI1723G). JARA is supported by the University of Glasgow School of Veterinary Medicine VetFund. We thank Lynn Stevenson, Frazer Bell and Lynn Oxford for technical assistance. PRM owns shares of Astra Zeneca. The rest of the authors declare no conflicts of interest.

## Figure legends

**Figure 1.** Replication kinetics of SARS-CoV-2 and HRV in ALI-cultures of HBECs. (A) SARS-CoV-2 titers in single SARS-CoV-2 infections (solid red line) and simultaneous SARS-CoV-2/HRV coinfections (dashed red line). (B) HRV titers in single HRV infections (solid cyan line) and simultaneous SARS-CoV-2/HRV coinfections (dashed cyan line). (C & D) SARS-CoV-2 (red) and HRV (cyan) titers in single infections (solid lines) and staggered SARS-CoV-2/HRV coinfections (dashed lines). The order of infections is described below each graph. SARS-CoV-2 is shown in red and HRV is shown in cyan.

**Figure 2.** MxA expression in ALI-cultures of HBECs. Representative images of MxA expression by fluorescence microscopy at various times post infection. ALI-cultures were mock infected, infected with SARS-CoV-2 only, HRV only, and coinfected with SARS-CoV-2 and HRV. Nuclei are colored in blue and MxA is colored in magenta. The scale bar indicates 50 μm.

**Figure 3.** Detection of SARS-CoV-2 in ALI-cultures of HBECs. Representative images of SARS-CoV-2 N detection by immunofluorescence in cells infected with SARS-CoV-2 (A); co-infected with SARS-CoV-2 and HRV (B); or mock infected (C). Nuclei are colored in blue and SARS-CoV-2 N protein is colored in red. The scale bar indicates 50 μm.

**Figure 4.** Replication kinetics of SARS-CoV-2 and HRV in ALI-cultures of HBECs coinfected simultaneously with SARS-CoV-2 and HRV in the presence or absence of BX795. (A) SARS-CoV-2 titers. (B) HRV titers. SARS-CoV-2 is shown in red and HRV is shown in cyan. Solid and dotted lines show infections in the presence of absence of BX795, respectively.

**Figure 5.** Reduction in COVID-19 growth rate for varying prevalence of rhinovirus infections in a given population and different assumptions for the duration of the refractory period. The growth rate in the absence of rhinovirus is assumed to be a 5% increase/day. Colors show the reduction in growth rate expressed as percentage.

Figure 1

Figure 2

Figure 3

Figure 4

Figure 5
