# Human Rhinovirus Infection Blocks Severe Acute Respiratory Syndrome Coronavirus 2 Replication Within the Respiratory Epithelium: Implications for COVID-19 Epidemiology

**DOI:** 10.1093/infdis/jiab147

**Published:** 2021-03-23

**Authors:** Kieran Dee, Daniel M Goldfarb, Joanne Haney, Julien A R Amat, Vanessa Herder, Meredith Stewart, Agnieszka M Szemiel, Marc Baguelin, Pablo R Murcia

**Affiliations:** 1 MRC–University of Glasgow Centre for Virus Research, Institute of Infection, Immunity and Inflammation, College of Medical, Veterinary and Life Sciences, University of Glasgow, Glasgow, United Kingdom; 2 School of Veterinary Medicine, College of Medical, Veterinary and Life Sciences, University of Glasgow, Glasgow, United Kingdom; 3 Imperial College London, London, United Kingdom

**Keywords:** SARS-CoV-2, rhinovirus, virus-virus interactions

## Abstract

Virus-virus interactions influence the epidemiology of respiratory infections. However, the impact of viruses causing upper respiratory infections on severe acute respiratory syndrome coronavirus 2 (SARS-CoV-2) replication and transmission is currently unknown. Human rhinoviruses cause the common cold and are the most prevalent respiratory viruses of humans. Interactions between rhinoviruses and cocirculating respiratory viruses have been shown to shape virus epidemiology at the individual host and population level. Here, we examined the replication kinetics of SARS-CoV-2 in the human respiratory epithelium in the presence or absence of rhinovirus. We show that human rhinovirus triggers an interferon response that blocks SARS-CoV-2 replication. Mathematical simulations show that this virus-virus interaction is likely to have a population-wide effect as an increasing prevalence of rhinovirus will reduce the number of new coronavirus disease 2019 cases.

## Teaser

Human rhinovirus triggers an innate immune response that blocks severe acute respiratory syndrome coronavirus 2 replication within human respiratory epithelium. Given the high prevalence of human rhinovirus, this effect might cause a population-wide reduction in new coronavirus disease 2019 infections.

The rapid spread of coronavirus disease 2019 (COVID-19) and its impact on global health highlights the importance of viral respiratory diseases. The human respiratory tract hosts a community of viruses that includes members of the Orthomyxoviridae (eg, influenza virus A and B), Pneumoviridae (eg, respiratory syncytial virus), Picornaviridae (eg, rhinovirus), Coronaviridae (eg, severe acute respiratory syndrome coronavirus 2 [SARS-CoV-2]), and other families [1, 2]. We and others have shown that interactions between cocirculating, taxonomically different respiratory viruses can influence patterns of infection [3, 4]. We showed that human rhinoviruses (HRVs) and influenza A viruses (IAVs) interact negatively at the individual patient and population level. It has also been postulated that the circulation of HRV delayed the spread of pandemic H1N1 influenza virus in France in 2009 [5]. Viral interference interactions at the host level are considered important in influencing observed population dynamics. Wu et al [4] demonstrated that HRV induces an interferon (IFN) response that protects against subsequent IAV infection in differentiated airway cultures, whereas Gonzalez et al [6] showed that RV attenuates influenza severity in a mouse model.

Nonpharmacological interventions have hampered our ability to determine the impact of SARS-CoV-2 on the epidemiology of respiratory viruses. However, it is possible that the emergence of SARS-CoV-2 will affect their ecology. Coinfection studies using air-liquid interface (ALI) cultures of differentiated respiratory epithelial cells can shed light on the nature of SARS-CoV-2 interactions with other viruses and their effect on virus replication. Here, we examined the replication kinetics of SARS-CoV-2 in the presence of HRV in the human respiratory epithelium. HRV was selected owing to (1) its high prevalence in the human population [7], (2) its negative interaction with IAV at the host and population level [3, 4], (3) its ability to induce a strong IFN response [4], and (4) the sensitivity of SARS-CoV-2 to IFN [8]. We used our experimental results as a proxy of within-host coinfection dynamics to simulate the impact of HRV circulation on the epidemiology of SARS-CoV-2 under different scenarios of HRV prevalence.

## METHODS

### Cells

Primary human bronchial epithelial cells (HBECs) were sourced from Epithelix Sarl. Cells were maintained and seeded on Transwell cell culture inserts (Falcon catalog no. 734-0036) using Epithelix human airway epithelial cell medium (Epithelix; EP09AM) and incubated at 37ºC with 5% carbon dioxide (CO_2_). An ALI was initiated once cells reached confluency, when the maintenance medium was switched to Pneumacult-ALI media (catalog no. 05001; STEMCELL Technologies). Vero E6 F5 cells were subcloned from Vero E6 cells, a gift from Michele Bouloy. A bulk population of Vero E6 cells was diluted in in Dulbecco’s minimum essential medium (DMEM) supplemented with 10% (vol/vol) fetal calf serum to 1 cell per 100 µL and plated into a 96-well format and incubated at 37ºC in a 5% CO_2_, humidified incubator. 

Wells were assessed for cell number with 0 and 3 cells per well observed. Once the population had expanded, each clonal population was further seeded into a single well in a 96-well plate. The next day, the plate was infected with 8400 plaque-forming units (PFUs) per well of SARS-CoV2 and left for 72 hours. The plates were fixed in 8% (wt/vol) formaldehyde in phosphate-buffered saline and stained with Coomassie brilliant blue (0.1% [wt/vol] Coomassie Brilliant Blue R-250; 45% [vol/vol] methanol; and 10% [vol/vol] glacial acetic acid) and assessed for cytopathic effect. Plates were scanned using a using the Celigo platform (Nexcelcom). Infection of 3 of 288 clones resulted in clearance of the monolayer (2H6, 5F3, and 6F5). 

These clones were further assessed for changes in plaque morphology and to determine whether the well-clearance assay generated representative titers. They were further assessed for growth characteristics. Two of the 3 clones were discarded owing to an underestimate of viral titer (2H6) and longer mean generation time of the cells (5F2) in comparison with the bulk population of Vero E6 cells. HeLa Ohio cells were a gift from Toby Tuthill (Pirbright Institute). Both cell lines were grown in DMEM with high-glucose and GlutaMAX (GIbco), and supplemented with 10% fetal bovine serum and 1% nonessential amino acids.

### Viruses

SARS-CoV-2 strain HCoV-19/England/02/2020 was sourced from Public Health England (GISAID accession no. EPI_ISL_407073) originating from a clinical isolate and was passaged twice in Vero E6 cells. HRV-A16 was sourced from the American Type Culture Collection (no. VR-283).

### Infection of HBEC Cultures

HBEC cultures were infected ≥35 days after ALI initiation. The apical surface of the cultures was washed twice with serum-free DMEM before infection (24 hours before and immediately before infection). Cells were inoculated with 10^4^ PFUs of either SARS-CoV-2 or HRV-A16, or a mixture containing 10^4^ PFUs of each virus, and incubated at 37ºC for 120 minutes. Previous experiments showed that inoculation of ALI cultures with 10 000 PFUs resulted in consistent replication of HRV and SARS-CoV-2 [9]. The inoculum was removed, and cultures were washed once. This wash was titrated by 50% tissue culture infectious dose (TCID_50_) assay and served as the 0-hour time point for growth curves. 

Cells were incubated at 37ºC with 5% CO_2_. At each time point, serum-free DMEM was added apically to each culture and incubated for 30 minutes at 37ºC. This was removed, aliquoted, and stored at −80ºC before subsequent titration. Each infection was carried out in 2 independent experiments and consisted of ≥3 technical replicates. Titrations of SARS-CoV-2 and HRV-A16 were performed on Vero E6 6F5 and HeLa Ohio cells, respectively. Virus samples were titrated in 10-fold serial dilutions in DMEM with 2% fetal bovine serum and 1% nonessential amino acids on confluent monolayers of cells. Each sample was titrated in triplicate. SARS-CoV-2 TCID_50_ plates were incubated at 37ºC, and HRV-A16 plates were incubated at 33^o^C. Plates were incubated for approximately 72 hours, fixed in 8% formaldehyde, and stained with 0.1% Coomassie Brilliant Blue. Cytopathic effect was recorded, and TCID_50_-per-milliliter titers were calculated using the Spearman and Kärber algorithm [10]. For BX795 experiments, ALI cultures were transferred to Pneumacult-ALI medium containing 6 μMol BX795 (or dimethyl sulfoxide) 18 hours before infection, with medium changed daily. All experimental infections were carried out under biosafety level 3 conditions.

### Tissue Processing and Immunostaining

After fixation in 8% formaldehyde for 16–24 hours, HBEC cultures were processed overnight for paraffin embedding, sectioned to 2–3-µm-thick sections, and mounted on glass slides. Two sections for each condition were sectioned and processed using pH 8 ethylenediaminetetraacetic acid antigen retrieval and permeabilized with 1% triton. 4′,6-diamidino-2-phenylindole (Thermo Fisher Scientific; catalog no. P36392) was included in the mounting medium, and slides were stained with primary sheep anti-N (nucleocapsid) immunoglobulin G antibody (DA114; mrcppu-covid.bio; 1:1000 dilution), primary mouse anti–myxovirus resistance protein A (MxA) antibody [11], primary mouse anti-VP2 antibody (QED Bioscience; no.18758), or a primary rabbit anti–human angiotensin-converting enzyme 2 (ACE2) antibody (Cell Signalling Technology). For immunofluorescence, primary antibodies were detected using an AlexaFluor 555-conjugated donkey anti-sheep antibody (A11015, Thermo Fisher Scientific; 1:1000 dilution) and an AlexaFluor 488-conjugated goat anti-mouse antibody (Sigma SAB4600056; 1:1000 dilution). For immunohistochemistry, anti–human ACE2 was detected using EnVision+ anti-rabbit horseradish peroxidase (Agilent K4003). Immunofluorescence sections were imaged using a Zeiss LSM880 confocal microscope, and immunohistochemistry sections using an Olympus BX51 microscope.

### Statistical Analysis and Data Visualization

Statistical analysis and data visualization were carried out using R 3.5.1 software [12]. Multivariable logistic regression models were used to investigate significance among the different conditions. Those models accounted for biological replicates, as this parameter was uneven, as well as treatment and time after infection. When biological replicate was not a significant parameter, this was removed to simplify the model. Models were run using the lme4 package [13]. Data visualization and figures were generated using the ggplot2 software package [14].

## RESULTS

**Figure 1.**
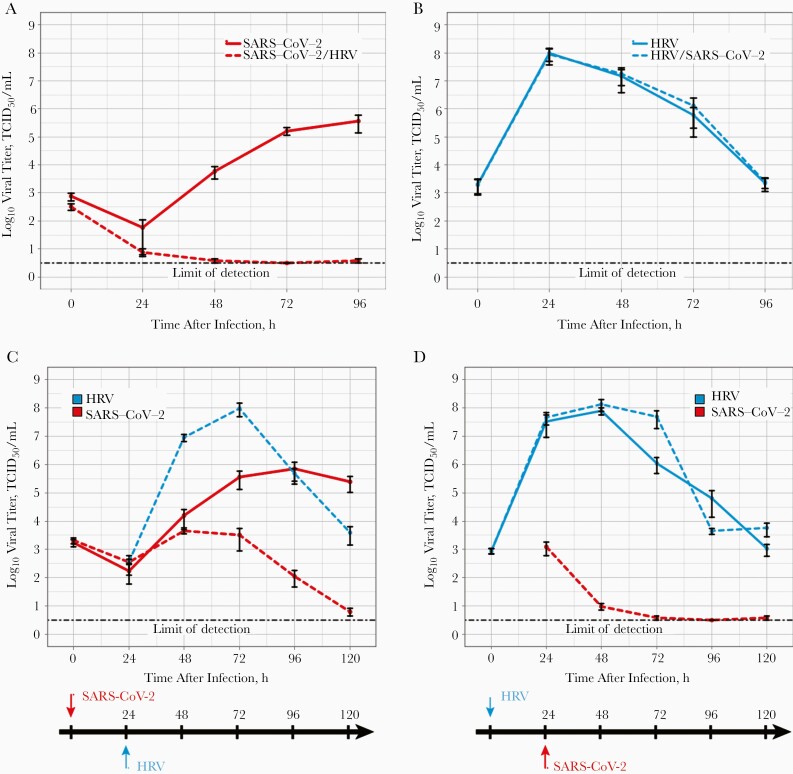
Replication kinetics of severe acute respiratory syndrome coronavirus 2 (SARS-CoV-2) and human rhinovirus (HRV) in air-liquid interface cultures of human bronchial epithelial cells. *A,* SARS-CoV-2 titers in single SARS-CoV-2 infections (*solid red line*) and simultaneous SARS-CoV-2/HRV coinfections (*dashed red line*). *B,* HRV titers in single HRV infections (*solid cyan line*) and simultaneous SARS-CoV-2/HRV coinfections (*dashed cyan line*). *C, D,* SARS-CoV-2 (*red*) and HRV (*cyan*) titers in single infections (*solid lines*) and staggered SARS-CoV-2/HRV coinfections (*dashed lines*). The order of infections is described below each graph. SARS-CoV-2 is shown in red, and HRV in cyan. Abbreviation: TCID_50_, 50% tissue culture infectious dose.

To determine if SARS-CoV-2 and HRV interact within the human respiratory epithelium, we infected ALI cultures of HBECs with SARS-CoV-2,with HRV, or with both viruses simultaneously. To assess the impact of coinfections on the replication kinetics of each virus, HRV and SARS-CoV-2 titers were determined at different times after infection from apical washes of coinfected cells and compared with their respective titers from single virus infections. SARS-CoV-2 exhibited highly contrasting replication kinetics in single infections and coinfections (*P* = .04) (Figure 1A). SARS-CoV-2 titers increased slowly from 24 hours after infection onward and up to 96 hours after infection in single infections, whereas in coinfections with HRV, SARS-CoV-2 titers decreased rapidly and were undetectable at 48 hours after infection (Figure 1A). In contrast, HRV titers displayed the same kinetics in single infections and coinfections: they increased rapidly during the first 24 hours, followed by a gradual and sustained decline (Figure 1B). 

Because simultaneous coinfections might not occur frequently during natural infection, we performed staggered coinfections of ALI-cultures of HBECs, as follows: cells were infected with HRV, and 24 hours later they were infected with SARS-CoV-2. This experiment was also repeated in the reverse order (ie, SARS-CoV-2 first, followed by HRV). As observed in simultaneous coinfections, SARS-CoV-2 growth was severely impaired in both staggered coinfections: when SARS-CoV-2 inoculation was followed by HRV infection (*P* = .03) SARS-CoV-2 replication increased between 24 and 48 hours after infection, as seen in SARS-CoV-2 single infection, but a subsequent sharp decrease was observed between 48 and 96 hours after infection (Figure 1C). When HRV inoculation was followed by SARS-CoV-2 infection, SARS-CoV-2 replication did not exceed the inoculum titer, and viral titers quickly declined (*P* = .006) (Figure 1D). In contrast, the growth of HRV was unaffected by SARS-CoV-2 (*P* = .20). regardless of the sequence order of infections (Figure 1C and D). When SARS-CoV-2 was inoculated first, the growth curve of HRV shifted and peaked at 72 hours after infection (Figure 1C), reflecting the delay in HRV inoculation. 

We tested whether the observed reduction of SARS-CoV-2 titers was due to a block in virus entry caused by HRV-induced down-regulation of the SARS-CoV-2 receptor, ACE2 [15]. To this end, we used immunohistochemistry to detect ACE2 in HRV or SARS-CoV-2 singly infected and coinfected epithelial cells. We observed high levels of ACE2 expression on the apical surface of the epithelium regardless of the infection status of the cells (Supplementary Figure 1), suggesting that HRV blocks SARS-CoV-2 infection via mechanisms that are independent of virus entry.

**Figure 2.**
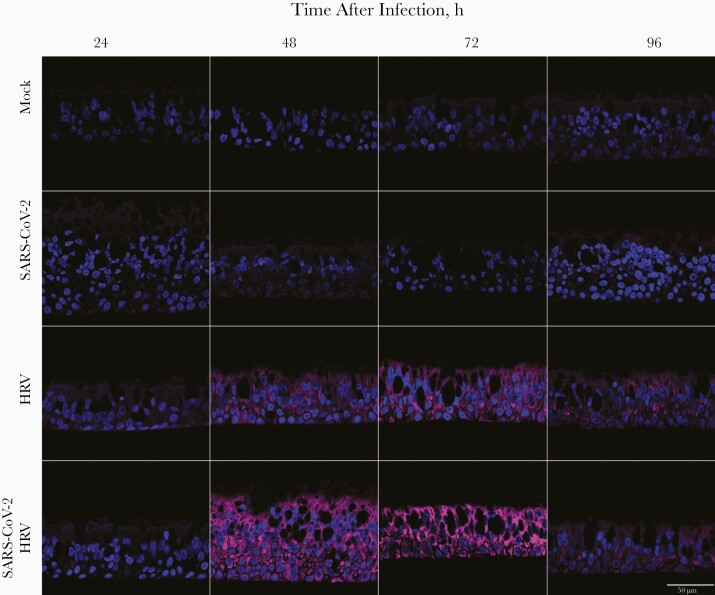
Myxovirus resistance protein A (MxA) expression in air-liquid interface (ALI) cultures of human bronchial epithelial cells, shown in representative images obtained with fluorescence microscopy at various times after infection. ALI cultures were mock-infected, infected with severe acute respiratory syndrome coronavirus 2 (SARS-CoV-2) only or human rhinovirus (HRV) only, or coinfected with SARS-CoV-2 and HRV. Nuclei are colored in blue, and MxA in magenta. Scale bar represents 50 μm.

SARS-CoV-2 is susceptible to IFN and encodes multiple genes that alter signaling pathways upstream and downstream of IFN production [8]. As HRV induces an IFN-mediated innate immune response that blocks IAV in ALI-cultures [4], we hypothesized that the observed block in SARS-CoV-2 replication was due to an HRV-triggered IFN response. To test this, we used fluorescence microscopy to examine the IFN-mediated innate immune activation induced by each virus. Specifically, we compared the in situ expression of MxA, a protein encoded by an IFN-stimulated gene that is highly up-regulated on IFN production [11]. Figure 2 shows that ALI cultures of HBECs infected with HRV express high levels of MxA, contrasting with the low levels of MxA observed in SARS-CoV-2–infected cultures. Coinfected cultures exhibited high levels of MxA expression, similar to those exhibited in single infections with HRV (Figure 2). 

**Figure 3.**
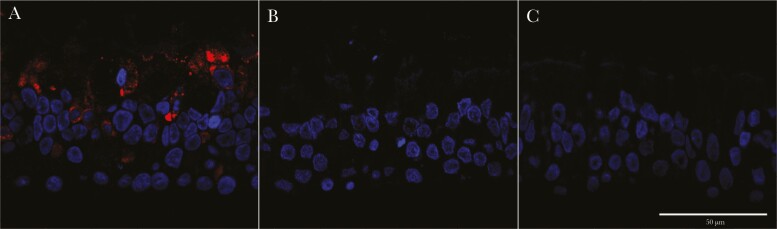
Detection of severe acute respiratory syndrome coronavirus 2 (SARS-CoV-2) in air-liquid interface cultures of human bronchial epithelial cells. Representative images show SARS-CoV-2 nucleocapsid detection by immunofluorescence in cells infected with SARS-CoV-2 (*A*); coinfected with SARS-CoV-2 and human rhinovirus (*B*); or mock-infected (*C*). Nuclei are colored in blue, and SARS-CoV-2 nucleocapsid protein in red. Scale bar represents 50 μm.

**Figure 4.**
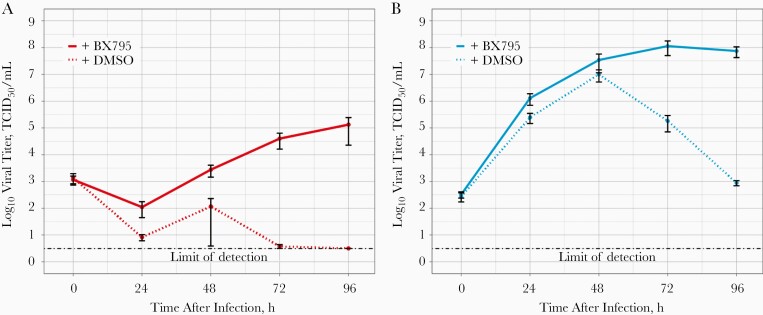
Replication kinetics of severe acute respiratory syndrome coronavirus 2 (SARS-CoV-2) and human rhinovirus (HRV) in air-liquid interface cultures of human bronchial epithelial cells coinfected simultaneously with SARS-CoV-2 and HRV in the presence or absence of BX795. *A,* SARS-CoV-2 titers. *B,* HRV titers. SARS-CoV-2 is shown in red, and HRV in cyan. Solid and dotted lines show infections in the presence or absence of BX795, respectively. Abbreviations: DMSO, dimethyl sulfoxide; TCID_50_, 50% tissue culture infectious dose.

We further performed immunofluorescence using antibodies directed against the nucleocapsid (N) of SARS-CoV-2 and observed that N expression is clearly detected mainly on the apical area of epithelial cells subject to single SARS-CoV-2 infection but undetectable in coinfected cells (Figure 3). Overall, our combined experiments confirmed (1) that SARS-CoV-2 replication within the ALI-cultures of HBECs does not progress in the presence of HRV and (2) that HRV triggers a faster and likely stronger IFN response compared with SARS-CoV-2. We therefore hypothesized that the block observed in SARS-CoV-2 replication was due to an innate immune response triggered by HRV. To test this, we performed HRV/SARS-CoV-2 coinfections in the presence of BX795, an inhibitor of TANK-binding kinase 1 that has been shown to block the IFN-mediated innate immune response in differentiated cultures of respiratory epithelium [4]. In the presence of BX795, the ability of SARS-CoV-2 to replicate in the respiratory epithelium is restored to levels comparable to those of SARS-CoV-2 single infection, despite the presence of HRV (Figure 4A). This confirms that the observed block in SARS-CoV-2 replication in coinfections with HRV was the result of negative interactions driven by the innate immune response induced by HRV. Interestingly, HRV replication was also increased in the presence of BX795, and titers plateau between 48 and 96 hours after infection, rather than declining, as observed in the dimethyl sulfoxide control coinfection and HRV single infection (Figure 4B). This indicates that virus-induced innate immune signaling also hampers HRV replication in HBECs.

**Figure 5.**
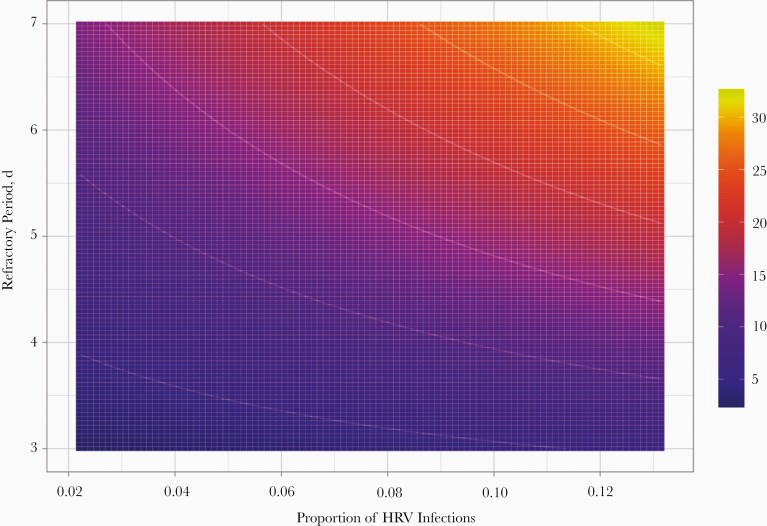
Reduction in coronavirus disease 2019 growth rate for varying prevalence of human rhinovirus (HRV) infections in a given population and different assumptions for the duration of the refractory period. The growth rate in the absence of rhinovirus is assumed to be a 5% increase per day. Colors show reductions in growth rate expressed as percentages.

Given the high prevalence of HRV, we wanted to determine whether the observed within-host interference could have an impact on the number of new COVID-19 cases in the population. We performed mathematical simulations, using the moment-generating function equation [16] to derive the change in the growth rate of SARS-CoV-2 infections as a result of having a fraction of the population refractory to COVID-19 owing to an episode of HRV infection (Data Analysis 1 in Supplementary Material). Our results show that the number of new SARS-CoV-2 infections decreases as the number of HRV infections increase, and this reduction increases with higher HRV prevalences and longer duration of the interference effect (Figure 5). When SARS-CoV-2 growth rates are low, HRV circulation can lead to SARS-CoV-2 infections not spreading, whereas exponential growth is expected in the absence of HRV.

## Discussion

Respiratory explants and ALI-cultures of human airway epithelium provide a highly controlled cellular environment that mimics to a considerable extent the natural site of infection and thus enables us to model the impact of virus tropism and innate immune responses on within-host infection dynamics [17]. Here we showed that HRV infection impairs SARS-CoV-2 replication and spread within the human respiratory epithelium. Our study shows that HRV exerts an indirect negative interaction, with a dominant inhibitory phenotype against SARS-CoV-2. Specifically, we showed that HRV triggers an IFN response that makes most cells nonpermissive to SARS-Cov-2 infection, while HRV is unaffected by the presence of SARS-CoV-2. The susceptibility of SARS-CoV-2 to the IFN response is illustrated by the number of genes present in its genome that are devoted to overcome the innate immune response (reviewed in [18]). 

We also showed that HRV hampers SARS-CoV-2 replication, even when the former was inoculated 24 hours after SARS-CoV-2. Overall, our results demonstrate that viral interference interactions induced by HRV infection can inhibit SARS-CoV-2 replication in the respiratory epithelium and builds on previous epidemiological, modeling, and experimental work on virus-virus interactions [3–5, 19]. Future studies to elucidate the molecular mechanisms of viral interference could enable us to wield virus-virus interactions to our advantage and use them as control strategies or therapeutic measures. For example, screening for HRV-induced genes with anti–SARS-CoV-2 activity might constitute a future research avenue to develop antiviral therapies against coronaviruses.

Recently, Wu et al [4] showed that the IFN response triggered by HRV also interferes with IAV replication. Our combined studies suggest that viruses that stimulate an IFN response in the respiratory epithelium might interfere with SARS-CoV-2 and IAVs. These findings have important implications, as they suggest that immune-mediated effects induced by mild, common cold virus infections, including HRV, might confer some level of protection against SARS-CoV-2, potentially attenuating the severity of COVID-19. Given the high transmissibility and prevalence of HRV, this effect might have an impact on the disease burden caused by COVID-19 at the population scale, with expected heterogeneities depending on HRV prevalence among different age groups. For example, this interference effect can contribute to differences in SARS-CoV-2 transmission between school-aged children (with high prevalence of HRV) and adult populations (with comparatively lower HRV prevalence).

Viruses are obligate intracellular pathogens that can infect only a restricted number of cell types within the body (a property known as *tropism*). Virus-virus interactions are likely to occur not only in the respiratory tract but also in other tissues that support multivirus environments, such as the gastrointestinal tract, where enteric infections are modulated by the gut virome [20] and also affect the immunogenicity of the live attenuated polio vaccine [21]. The nature of such interactions (ie, positive, negative, or neutral) is largely unknown and likely to be influenced by the specific viruses involved, the timing of each infection, and the interplay between the host’s response to each virus.

There is a vast body of knowledge on the impact of evolution on virus-host interactions [22–25]. Many studies have focused on the evolutionary arms race between viruses and hosts, where the host’s immune system evolves antiviral mechanisms to stop viral replication and viruses evolve to evade antiviral proteins. We propose that virus-virus interactions influence this arms race and contribute to shaping their molecular interplay. For example, it is feasible to think that HRV infections in humans might be mutually beneficial: from an HRV perspective, humans evolved a tightly regulated immune response that allows HRV to replicate and transmit while it blocks other potentially competing viruses. From a host’s perspective, HRV infections, which are usually associated with mild disease, stimulate an antiviral response that prevents infections by more severe (and sometimes lethal) viruses, such as SARS-CoV-2 and IAV. Future studies using coinfections are needed to shed light on the role of ecology and evolution on virus-virus interactions and their impact on virus host range, transmission and disease.

## Supplementary Data

Supplementary materials are available at *The Journal of Infectious Diseases online*. Consisting of data provided by the authors to benefit the reader, the posted materials are not copyedited and are the sole responsibility of the authors, so questions or comments should be addressed to the corresponding author.

jiab147_suppl_Supplementary_Data

jiab147_suppl_Supplementary_Figure_1

## Notes

***Acknowledgments*.** We thank Lynn Stevenson, Frazer Bell, and Lynn Oxford for technical assistance.

***Financial support.*** This work was supported by the Medical Research Council of the United Kingdom (grants MR/R502327/1 to D. M. G., MR/N013166/1 to J. H., and MC_UU_12014/9 to P. R. M.); the Medical Research Council under a concordat with the UK Department for International Development and the National Institute for Health Research Health Protection Research Unit in Modelling Methodology (funding to M. B.); the German Research Foundation (Deutsche Forschungsgemeinschaft; project 406109949 to V. H.); the Federal Ministry of Food and Agriculture (Förderkennzeichen; grant 01KI1723G to V. H.); and the University of Glasgow School of Veterinary Medicine VetFund (J. A. R. A.).

***Potential conflicts of interest.*** P. R. M. owns shares of AstraZeneca. All other authors report no potential conflicts. All authors have submitted the ICMJE Form for Disclosure of Potential Conflicts of Interest. Conflicts that the editors consider relevant to the content of the manuscript have been disclosed.
