# Supplementary material for: Human Rhinovirus Infection Blocks Severe Acute Respiratory Syndrome Coronavirus 2 Replication Within the Respiratory Epithelium: Implications for COVID-19 Epidemiology
Source: J Infect Dis. 2021 Mar 23;224(1):31–8. doi: 10.1093/infdis/jiab147 (PMC8083659; doi:10.1093/infdis/jiab147)
Supplement: jiab147_suppl_Supplementary_Data [file jiab147_suppl_supplementary_data.pdf]

Supplementary Data

Data analysis S1

Potential impact of rhinovirus prevalence on the growth rate of SARS-CoV-2 at the population level

Theoretical derivation

Let α be the prevalence of human rhinovirus (HRV) infections in the studied population. We can then derive α^* the prevalence of SARS-CoV-2 refractory cases as a function of the mean duration of the refractory period t_r:

$$\alpha^* = \frac{t_r}{t_s}\alpha$$

with t_s the mean duration of the symptoms.

The presence of some individuals refractory to SARS-CoV-2 infection will have an impact on the the effective reproduction of SARS-CoV-2. Compared with the basic reproductive number R_0 in the absence of refractory cases linked with past HRV infections, assuming that refractory cases are fully protected from infection by SARS-CoV-2, the new effective reproductive number becomes

$$R_e^{cov|rhino} = (1-\alpha^*)R_0.$$

Assuming an exponential distribution for the generation interval, we can derive the growth rate of SARS-CoV-2 cases for a given prevalence of HRV infections using the moment generating function equation from (Wallinga and Lipsitch 2007):

$$r_{cov|rhino} = \frac{1}{t_c}\left(R_e^{cov|rhino} - 1\right)$$

with $r_{cov|rhino}$ the growth rate in the presence of rhinovirus and t_c the mean of the generation interval for SARS-Cov-2.

Combining with the previous equation we get:

$$\begin{aligned} r_{cov|rhino} &= \frac{1}{t_c}((1-\alpha^*)R_0 - 1) \\ &= \frac{1}{t_c}((1-\alpha^*)(1+rt_c) - 1) \\ &= r(1-\alpha^*) - \frac{\alpha^*}{t_c} \end{aligned}$$

with r is the growth rate of SARS-Cov-2 in the absence of Rhinoviruses.

Interpretation

The presence of HRV-induced refractory cases leads to a reduction in the growth rate of SARS-CoV-2 infections at the population level. The effect increases with the duration of the refractory period and is also influenced by the generation interval of SARS-CoV-2 in the human population. For a small value of growth rate, the presence of HRV can lead to a negative growth rate, meaning that SARS-CoV-2 infections cannot take off in the population. In contrast, would have grown exponentially in the absence of HRV. Fig. 1 shows how the growth rate of SARS-CoV-2 infections decreases linearly as a function of the prevalence of refractory cases in the population.

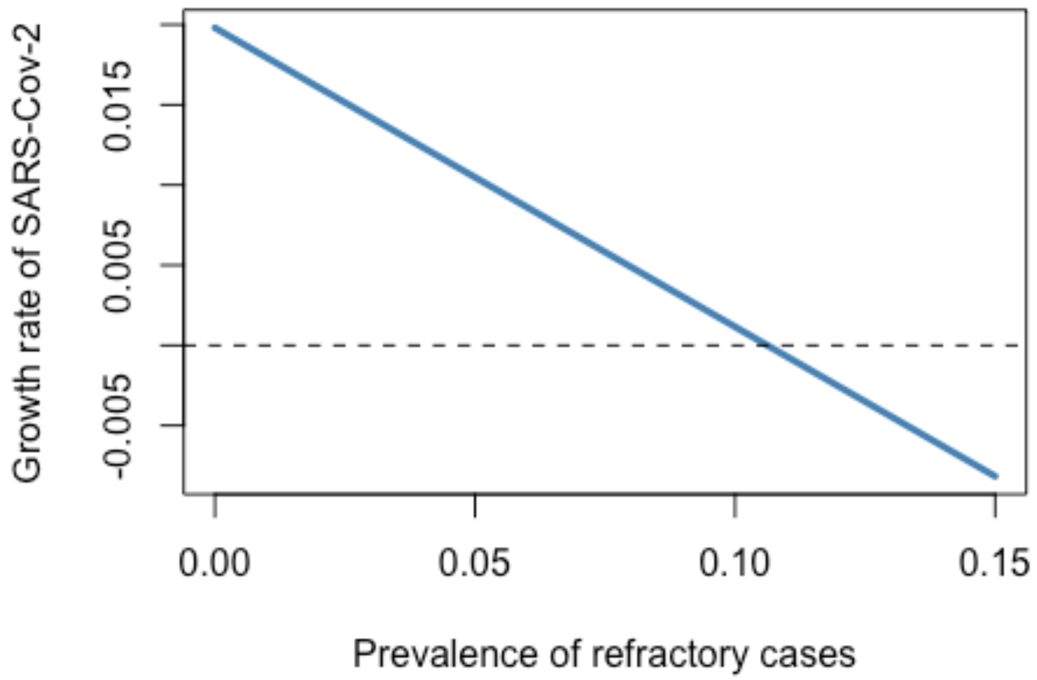

Figure 1. Growth rate of SARS-CoV-2 infections in a human population with co-circulation of HRV leading to "refractory" individuals with short-term protection. Growth rate in the absence of rhinovirus is assumed to be 2% per day. We assume a mean generation time of SARS-CoV-2 infections of six days.

Example of impact

Assuming that the duration of common cold symptoms is eight days (consistent with (Royston and Tapparel 2016)), a refractory period of seven days, and 5% of the population display HRV-related symptoms.

A growth rate of 5% per day corresponds to a growth rate of:

$$r = log(1.05) \approx 0.04879016$$

.

The growth rate in the presence of HRV is thus:

$$r_{cov|rhino} = log(1.05) * (1 - 0.05 * 7/8) - 0.05 * 7/(8 * 6) = 0.03936393$$

which can be expressed in percentage of increase per day:

$$e^{0.03936393} = 1.040149$$

so a 4% increase per day, corresponding to a reduction of 19.7%.

Figure 2 shows the impact expressed as a percentage reduction of the "base" growth rate as a function of the proportion of HRV infections in the studied population and the duration of the refractory period in days. For a growth rate of SARS-CoV-2 infections of 5%/day in the absence of HRV, a prevalence of 13% of HRV infections would result in a reduction of 33% of the growth rate for SARS-CoV-2 infections in the affected population if the refractory period is seven days.

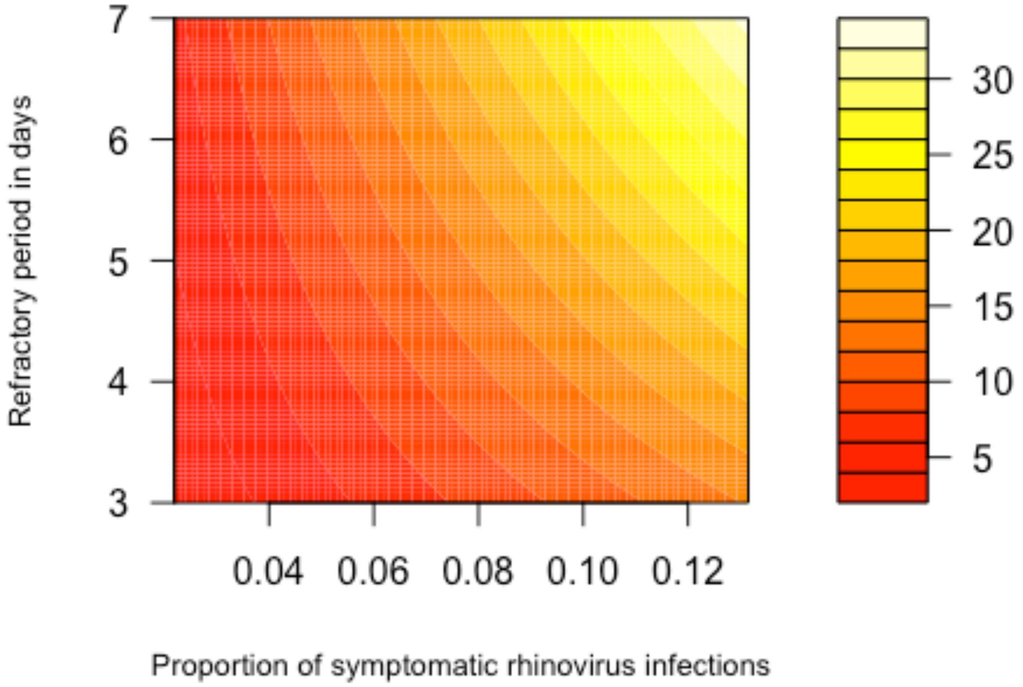

Figure 2. Reduction in growth rate for various prevalence of HRV infections in a given population and different assumptions for the duration of the refractory period. "Base" growth rate in the absence of HRV is assumed to be at a 5% increase per day. Colors show the reduction in growth rate expressed as percentage.

Royston, L, and C Tapparel. 2016. “Rhinoviruses and Respiratory Enteroviruses: Not as Simple as Abc.” *Viruses*. U.S. National Library of Medicine. https://pubmed.ncbi.nlm.nih.gov/26761027/.

Wallinga, J, and M Lipsitch. 2007. “How Generation Intervals Shape the Relationship Between Growth Rates and Reproductive Numbers.” *Proceedings Biological Sciences*, February. The Royal Society. https://www.ncbi.nlm.nih.gov/pmc/articles/PMC1766383/.
