# Supplementary material for: Human Rhinovirus Infection Blocks Severe Acute Respiratory Syndrome Coronavirus 2 Replication Within the Respiratory Epithelium: Implications for COVID-19 Epidemiology
Source: J Infect Dis. 2021 Mar 23;224(1):31–8. doi: 10.1093/infdis/jiab147 (PMC8083659; doi:10.1093/infdis/jiab147)
Supplement: jiab147_suppl_Supplementary_Figure_1 [file jiab147_suppl_supplementary_figure_1.pdf]

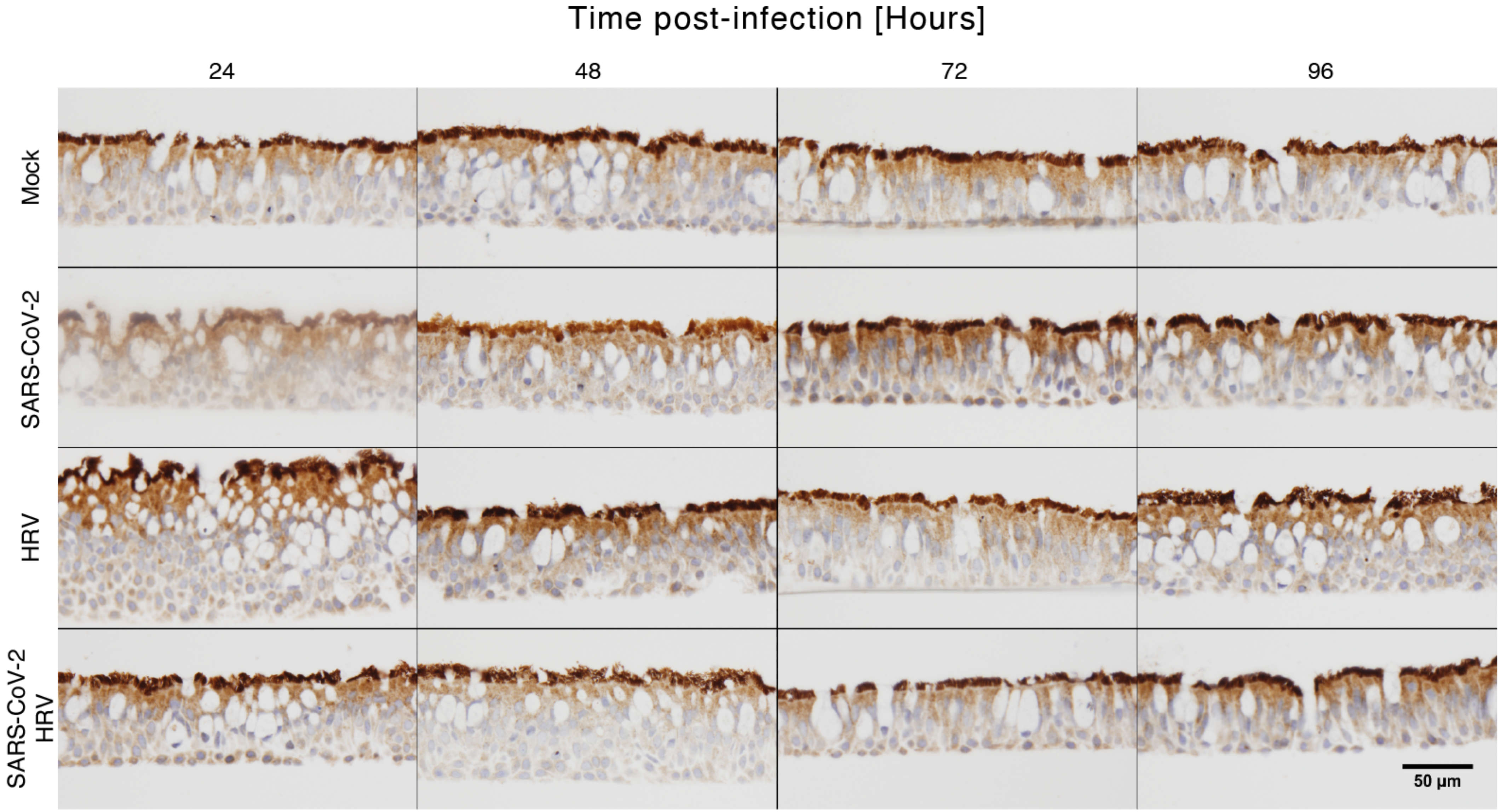

Supplementary Figure 1. ACE2 expression in ALI-cultures of HBECs. Representative light microscopy images of ACE2 expression detected by immunohistochemistry at various times post infection. ALI-cultures were mock infected, infected with SARS-CoV-2 only, HRV only, and coinfected with SARS-CoV-2 and HRV. ACE2 is colored in brown. The scale bar indicates 50 μm.
